# Telemedicine and Virtual Reality at Time of COVID-19 Pandemic: An Overview for Future Perspectives in Neurorehabilitation

**DOI:** 10.3389/fneur.2021.646902

**Published:** 2021-03-25

**Authors:** Marta Matamala-Gomez, Sara Bottiroli, Olivia Realdon, Giuseppe Riva, Lucia Galvagni, Thomas Platz, Giorgio Sandrini, Roberto De Icco, Cristina Tassorelli

**Affiliations:** ^1^Department of Human Sciences for Education “Riccardo Massa,” Center for Studies in Communication Sciences “Luigi Anolli” (CESCOM), University of Milano-Bicocca, Milan, Italy; ^2^Faculty of Law, Giustino Fortunato University, Benevento, Italy; ^3^Headache Science and Neurorehabilitation Center, Istituto di Ricovero e Cura a Carattere Scientifico Mondino Foundation, Pavia, Italy; ^4^Department of Psychology, Catholic University of Milan, Milan, Italy; ^5^Applied Technology for Neuro-Psychology Laboratory, Istituto Auxologico Italiano, Istituto di Ricovero e Cura a Carattere Scientifico, Milan, Italy; ^6^Center for Religious Studies, Bruno Kessler, Foundation, Trento, Italy; ^7^BDH-Klinik Greifswald, Institute for Neurorehabilitation and Evidence-Based Practice, “An-Institut,#x0201D; University of Greifswald, Greifswald, Germany; ^8^Neurorehabilitation Research Group, University Medical Centre Greifswald (UMG), Greifswald, Germany; ^9^Department of Brain and Behavioral Sciences, University of Pavia, Pavia, Italy

**Keywords:** telemedicine, healthcare, virtual reality, neurorehabilitation, COVID-19

## Abstract

In catastrophic situations such as pandemics, patients' healthcare including admissions to hospitals and emergency services are challenged by the risk of infection and by limitations of healthcare resources. In such a setting, the use of telemedicine interventions has become extremely important. New technologies have proved helpful in pandemics as a solution to improve the quality of life in vulnerable patients such as persons with neurological diseases. Moreover, telemedicine interventions provide at-home solutions allowing clinicians to telemonitor and assess patients remotely, thus minimizing risk of infection. After a review of different studies using telemedicine in neurological patients, we propose a telemedicine process flow for healthcare of subjects with chronic neurological disease to respond to the new challenges for delivering quality healthcare during the transformation of public and private healthcare organizations around the world forced by COVID-19 pandemic contingency. This telemedicine process flow represents a replacement for in-person treatment and thereby the provision equitable access to the care of vulnerable people. It is conceptualized as comprehensive service including (1) teleassistance with patient counseling and medical treatment, (2) telemonitoring of patients' health conditions and any changes over time, as well as (3) telerehabilitation, i.e., interventions to assess and promote body functions, activities, and consecutively participation. The hereby proposed telemedicine process flow could be adopted on a large scale to improve the public health response during healthcare crises like the COVID-19 pandemic but could equally promote equitable health care independent of people's mobility or location with respect to the specialized health care center.

## Introduction

On 20 March 2020, the World Health Organization (WHO) declared the pandemic state due to the spread of SARS-CoV-2 (1). In December 2020, more than 72 million subjects had been diagnosed with COVID-19 over the world, and more than 1.5 million of them had died (2). Due to the lack of an effective medical treatment to fight against the SARS-CoV-2, the healthcare measures mainly focused on symptomatic treatment, social distancing, use of device for individual protection, and the mandatory quarantine after being in close contact with an infected person (2). In this setting, medical visits, non-urgent treatments, and non-urgent medical issues, particularly for vulnerable subjects such as persons with neurological disorders, were initially interrupted and then re-assumed but frequently with a reduced scope. These measures have inevitably created long waiting lists and delays on medical visits, thus ultimately affecting patients' quality of life. Nevertheless, some preliminary efforts for maintaining the standard of care in the field of neurorehabilitation have been proposed (3, 4). Notably, the pandemic has also posed ethical questions for the healthcare system and the clinicians themselves (5). For instance, doctors had to face the dilemma of who can be treated at the hospital or at home, or who can be admitted to the limited number of beds in the intensive care units (ICU). Ultimately, in some areas, the most difficult question became how to fairly distribute scarce life-supporting clinical resources with implications for COVID-19 survivals. People with a severe chronic neurological condition who depend on a caregiver for their needs and/or to carry out their daily life routine had to face a difficult situations during the pandemic (5). COVID-19 is particularly lethal for the elderly with pre-existing conditions such as neurodegenerative and neuropsychiatric disorders, as they are a vulnerable population needing continuous supervision (6–8).

In times of stressed healthcare resources, the public health guidelines endorse the priority of treatment to those who are at short-term risk of death (9, 10). Moreover, the argument exists that young people should have priority over elderly people, even though whether and how this rule should be implemented is still controversial (10, 11). It must be noted that only a minority of people testing positive for SARS-CoV-2 become severely ill (12). Most people affected by COVID-19 present with mild symptoms and recover over a few days or weeks. From a healthcare point of view, this situation demands new ways to monitor the clinical situation of a large number of subjects at home. In addition, COVID-19 persons may develop a post-intensive care syndrome, presenting motor, cognitive, and emotional disorders, requiring an intensive rehabilitation program and a long-term supervision (13, 14). In patients with neurological disorders, the chronic persistence of COVID-19 have led to re-organized neurorehabilitation services accordingly (15, 16). In this regard, the use of new telecommunication technologies integrating telemedicine systems represents an alternative solution to facilitate the exchange between the healthcare providers and the patients (17, 18). Recently, some investigations reported the effectiveness of telemedicine services in remotely assisting, monitoring, and treating COVID-19 subjects or other diseases (17, 19–27). Indeed, a well-organized network could have the potential to reduce case fatality or at least provide a better management and supervision of the clinical conditions of vulnerable patients, such as those with neurological disorders, during the COVID-19 pandemic (28).

In this perspective article, we propose a telemedicine process flow representing a viable alternative to respond to the new challenges for patient care forced by the transformation of public and private healthcare organizations due to the COVID-19 pandemic. This network will represent a replacement for in-person treatment, providing equitable access to care for vulnerable people, including subjects with chronic neurological disorders. Such a network, which can be time- and cost-saving in normal situations, may feature two important added values: (1) safety and (2) access to care for a wider number of subjects.

## Telemedicine at the Time of COVID-19

In the ‘70s, Thomas Bird introduced the term “telemedicine,” meaning “healing at a distance,” which implies the delivery of healthcare services by using telecommunication technologies (29–31). Specifically, telemedicine interventions aim to facilitate healthcare treatment, limiting or avoiding hospitalization (29). More recently, WHO described “telemedicine” or “e-Health” as the use of technology related to informatics and telecommunication, i.e., information and communication technologies (ICT), directed to provide a positive effect in the patient’s health status (32). The main goals of telemedicine are to (1) improve the access to health care for rural areas, (2) give the physicians better access to tertiary consultation, (3) allow physicians to conduct remote examinations, (4) reduce health-care costs, (5) provide health-care services to a larger geographic region and or population, (6) reduce the need to transfer patients to the care centers, and (7) improve patient care (33).

During the COVID-19 pandemic, telemedicine represents an additional solution for healthcare services, allowing to deliver them directly at patient's home, reducing risks of possible infections, and enabling virtual triage to mitigate the negative psychological effects of social isolation (34). Then, with the current limitations in assisting patients at the hospital, the use of new telecommunication technologies by means of integrating telemedicine systems into the clinical routine may facilitate the maintenance of the remote relationship between healthcare providers and neurological patients (17, 35, 36). In this framework, the concept of “telemedicine” involves three treatment categories allowing to assist, monitor, and counseling patient remotely: (1) tele-assistance, (2) telemonitoring, and (3) telerehabilitation (37, 38). In the following section, we will discuss the three concepts embedded in the “telemedicine” overarching concept.

### Tele-Assistance

The concept of tele-assistance refers to the use of new technologies for patients' counseling at a distance. There are different modalities for providing tele-assistance: video-conferencing, e-mail, on-line chat sessions, forums, telephone calls, and mobile phone messages (39). A large number of studies have demonstrated the effectiveness of tele-assistance when dealing with patients with chronic disorders, such as cancer (40), diabetes (41), chronic respiratory failure (42), cystic fibrosis (43), brain injury (44), chronic pain (45), and stroke (46). For instance, a recent study demonstrated the effectiveness of tele-assistance at improving quality of life in people suffering from neuromuscular diseases (39). In this study, 24 participants with neuromuscular diseases were assisted through video-conferencing sessions in an on-line psychosocial program lasting 3 months. Participants reported benefits in some psychosocial variables as “getting along with people,” “psychosocial domain,” and “life activities” when compared to a control group (39). Others used a tele-assistance integrated care intervention to monitor patients with amyotrophic lateral sclerosis by using telephone calls, showing important time- and cost-saving benefits (47). One of the most useful features of tele-assistance is the possibility to support patients comprehensively from symptom onset to medical treatment delivery. For instance, a tele-assistance protocol—consisting in phone and video-conferencing connection between the ICU ambulance and the clinicians at the hospital—reduced the waiting time from symptom onset to treatment delivery in patients with stroke (48). This approach could be particularly useful at the time of the COVID-19, allowing the clinicians to assist and counsel patients at a distance, sending the clinical staff for the treatment delivery directly at their homes if and when necessary, thus avoiding the presence of the patients at the hospital.

### Telemonitoring

The concept of telemonitoring is defined as the use of information provided by the technology to monitor the patient's health state at a distance (49, 50). Telemonitoring systems are promising approaches able to reduce clinical complications in chronic patients (49), as in case of neurological disorders. For instance, it has been effectively used in patients with neuromuscular diseases (51, 52) and multiple sclerosis (MS) (53). Telemonitoring systems consist in the biometric tracking and transmission to the clinicians of physiological and/or behavioral data of the patients (e.g., heart rate, breathing rate, gait pattern, motor functions, etc.) in synchronous or asynchronous videoconferencing (54). Telemonitoring has also been proposed to deliver new data necessary for differential diagnosis or to stage illnesses in a health telematic network (55). Recently, telemonitoring has been used in patients with confirmed or suspected COVID-19 remotely, allowing for the timely identification of worsening symptoms (56). This approach seems particularly useful for telemonitoring COVID-19 patients with other chronic or high-risk pathologies (e.g., multiple sclerosis, Parkinson's disease, and myasthenia gravis) as it would limit the number of hospitalizations, optimize healthcare resources, and reduce the risk of virus transmission.

### Telerehabilitation

Telerehabilitation (TR) is a young telemedicine subfield consisting in the use of new telecommunication-based practices for controlling and conducting rehabilitation at a distance (57). TR can be used in all those situations in which the patient and the therapist cannot be in the same location. TR allows to begin the rehabilitation process as soon as possible after hospital discharge and increases the care access to individuals who are home-forced or geographically remote from their healthcare service (58–60). Hence, TR-based systems represent solid solutions to treat patients with an alternative way compared to the traditional face-to-face approach (58), providing benefits for the healthcare system and patients in terms of cost-effectiveness and feasibility for large-scale implementations. To this end, TR can use different types of technologies, such as sensor-based technology, tele/video-conference, specific *ad hoc* software, or virtual reality (61). Moreover, it has been shown that through telerehabilitation systems it is possible to foster patient motivation and participation in their own rehabilitation process (62), thus improving their well-being (63). TR may be useful for the treatment of motor, cognitive, or psychological deficits. Preliminary evidence indeed suggests its application in stroke, cerebral palsy, traumatic brain injury, multiple sclerosis (MS), and Parkinson's disease (PD), in particular as for treating motor- and speech-related impairments (54–58). TR has also been used for cognitive deficits (64) associated to neurological diseases, such as stroke, MS, brain tumors, Alzheimer's disease, and mild cognitive impairment (60–63, 65, 66).

In line with the necessary adaptation of healthcare services to the COVID-19, TR technological solutions are increasingly considered as potentially effective options for continuing the rehabilitation process at a distance (45, 67–70). Currently, many efforts are now focused on the treatment of subjects recovering from COVID-19 (71–74), but it seems extremely important to implement TR protocols also in non-COVID subjects in various settings of neurological care, in order to provide a continuity of care during this pandemic contingency and possibly in the future (75–78). During the COVD-19 pandemic, we have tested an innovative TR approach for the remote treatment of cognitive deficits in neurodegenerative diseases (79, 80) called HomeCoRe (Home Cognitive Rehabilitation) (81). HomeCoRe is a patient-tailored intervention stimulating many cognitive abilities, which is the home-based version of a previously tested computer-based cognitive training program (CoRe) (82–85), devised for the hospital setting. The system proved useful for providing continuity of care after hospital discharge in a condition of safety and distance and thus can be incorporated into clinical routine protocols.

## Technological Solutions for Telemedicine

Even though telemedicine interventions clearly have limitations compared to a hands-on approach in medicine (86, 87), the development of new technologies has also advantages over face-to-face health care, e.g., it allows the clinicians to follow the patients in a synchronous or asynchronous way. Synchronous telemedicine refers to the intervention performed in real time through a video call that can be conducted through a smartphone or a webcam connected to the computer (88). Asynchronous telemedicine interventions refer to the “store-and-forward” technologies, which allow monitoring and collection of physiological and/or behavioral data through wearable or implantable devices connected to an online or virtual platform and then sending the information to a clinical center for review and consultation (88, 89). The most common technological solutions used to provide telemedicine interventions are smartphones, tablets, and wearable sensors (90), including digital applications for self-exercises or monitoring the behavioral or physiological state of the patients (91). However, in the last 20 years, some telemedicine interventions have integrated the use of virtual reality (VR) platforms to deliver personalized rehabilitation training or clinical interventions at a distance (92–94). In some instances, VR can provide full-immersed virtual environments where the patient can feel present (being there) inside the virtual environment (95, 96). In the proposed process flow, VR can be used as an advanced communication interface, in which the patient can interact with different sensory information coming from different modalities, while performing specific rehabilitation tasks within the VR environment. VR systems enable a more intuitive mode of interacting with information, for the clinicians and the patients (63, 92, 97–100).

One of the main advantages of VR is that, through the use of virtual avatars, it is possible to induce virtual body ownership illusions toward the virtual body (physical possession of the virtual body) (101). During the last years, some investigations attempted to use virtual body ownership illusion for rehabilitation purposes in chronic patients (102–111). Some investigations proposed the integration of virtual body ownership illusions within a VR training for telemedicine purposes (100, 112). However, to the best of our knowledge, a comprehensive integrated telemedicine platform that provides synchronous and asynchronous interventions by means of VR, virtual body ownership illusions, and wearable sensors for real-time telemonitoring has neither been created nor tested. In the next paragraph, we propose an integrated telemedicine system for assisting, monitoring, and treating subjects with chronic neurological diseases during this pandemic situation and beyond.

## A New Telemedicine Network for Neurorehabilitation During Covid-19 Pandemic

Telemedicine services have the potential to provide medical service at a distance and in some instances even to save lives, while allowing patients and clinicians to be in touch safely (113). For this reason, many public health systems worldwide have been seeking for qualified and certified digital medical services to provide a continuity of care at a distance (114). However, most of the countries were unprepared for managing patients with a modern digital approach (113, 115–117). To facilitate the process, at the beginning of 2020, the American Medical Association wrote a telehealth implementation playbook with the definitions of “telehealth” or “telemedicine” as follows: (1) real-time video-conferencing between the patients and the clinicians being in different locations; (2) image and data collection stored and forwarded for the later data interpretation; (3) remote patient's monitoring through the use of mobile health tools, wearable sensors, and devices; and (4) virtual checks through phone calls, messaging, or videoconferencing (118). It must be noted that the definition did not include motor or cognitive rehabilitation based on digital platforms during and beyond the COVID-19.

**Figure 1:**
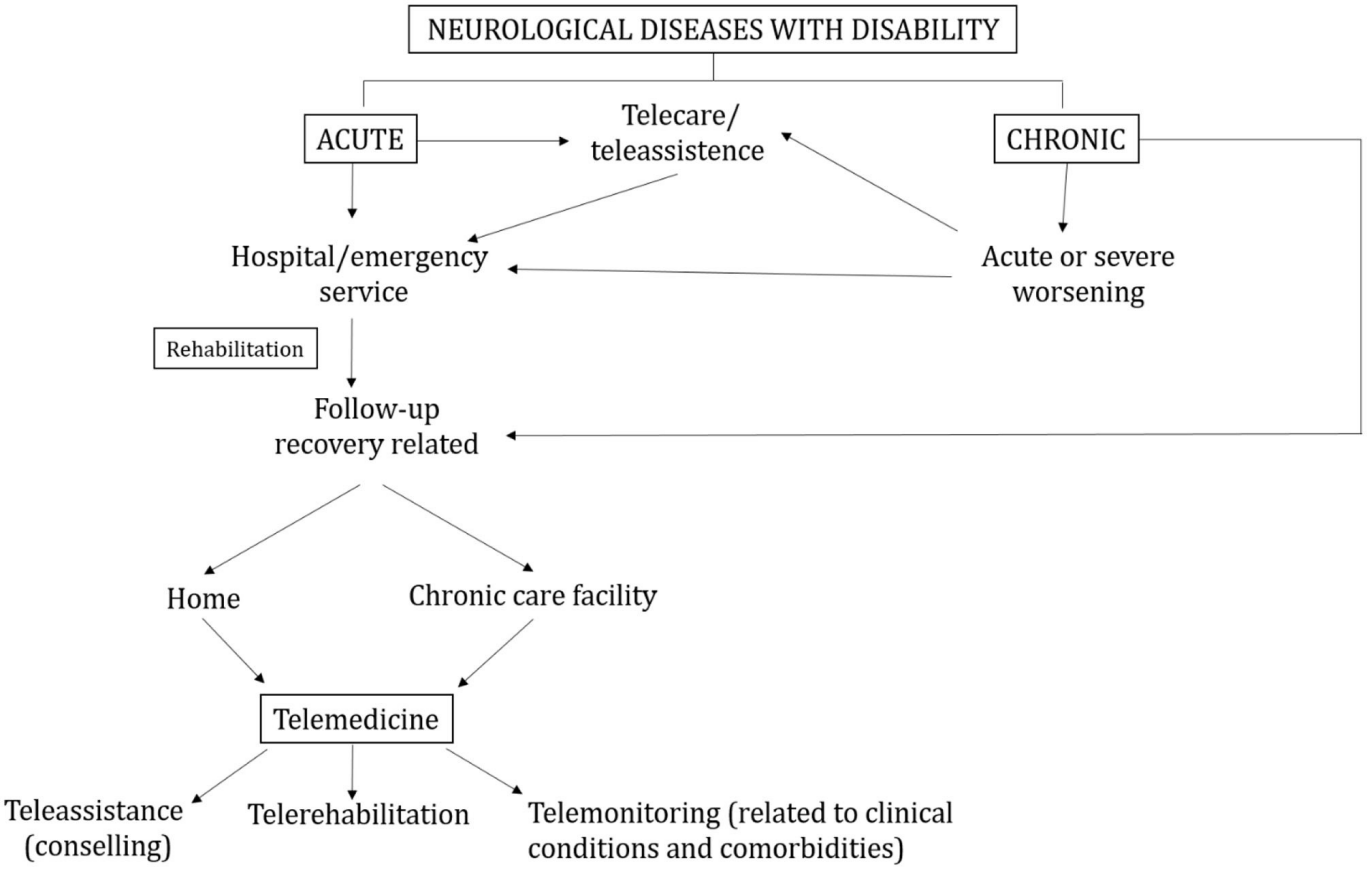
Flow chart of the clinical and telemedicine pathways in patients with acute or chronic neurological diseases.

Based in the above-commented literature and after a review of different studies using telemedicine for remote monitoring and intervention in patients with neurological disorders, here, we propose a telemedicine process flow for remotely managing patients with neurological disorders by including the following components: (1) tele-assistance or patient counseling: weekly or monthly videoconferencing with a health care provider that is tailored for the patient disorder; (2) telerehabilitation: reminder and performance of physical, communicative, and/or cognitive rehabilitation assessment and training through the digital platform; (3) telemonitoring: remote monitoring of the behavioral or physiological responses through the wearable sensors connected to the digital platform; (4) interpretation of stored data by the clinicians; and (5) virtual follow-up: virtual checks between the patients and the healthcare provider for adjusting the healthcare routine based on data interpretation. All these components will create a closed-loop telemedicine process flow, where the clinicians are enabled to visit and monitor a large number of patients with a virtual face-to-face approach through videoconferencing, thus reducing the need of transportation (of people with mobility restrictions) and avoiding the risk of infection on both ends in case of particular emergencies (Figure 1). Moreover, the telemedicine process flow can facilitate the active involvement of both the patients themselves and their caregivers in the healthcare process, which is a crucial element when dealing with telemedicine solutions for managing vulnerable populations in need of continuous supervision. The proposed telemedicine process flow would also enable clinicians to detect early sign or symptoms of COVID-19. The telemedicine process flow should be based on easy-to-use and accessible technology such as smartphones or tablets, integrated with a VR platform to conduct the healthcare routine. The same devices could also be used for telemonitoring patients' physiological or behavioral responses. Even though the proposed telemedicine intervention would be very helpful for managing patients during the COVID-19 pandemic, this telemedicine process flow can be also applied in normal circumstances avoiding or reducing patients' need for transportation or hospitalization and allowing clinicians to follow their patients at a distance, where in-person evaluations can be also considered as a complement of the telemedicine intervention. This could implement patients' engagement and activation (119).

## Limitations

Even though the proposed telemedicine intervention can be an optimal solution for monitoring and treating patients at a distance during a pandemic situation, the proposed telemedicine process flow still presents some limitations. A limitation is the availability of easy-to-use technology to deliver tele-consultations or for monitoring the patient's behavioral or physiological responses. Such limitations have been also suggested in earlier studies (36). Moreover, the performance of the training routine alone can represent some risk for the patients such as patient's falls or a bad performance of the exercises. Further, still, there is a lack of standardized tools to be used for virtual reality training and remote monitoring.

## Conclusion

The COVID-19 pandemic has created new challenges to patient care, imposing adaptation of healthcare facilities. ICT can be extremely useful in this adaptation process and also to maintain people connected with the world (120). These adaptations should be extended to the delivery of care for neurological diseases. Here, we proposed a telemedicine process flow for healthcare of subjects with chronic neurological disease. In the future, this telemedicine process flow could be implemented and applied on a large scale not only to improve the public health capacity and to allow clinicians to deliver good quality care in case of particular emergencies such as COVID-19 but also to provide equitable health care for patients with mobility restrictions or living remotely from specialized health care centers. Even though the proposed telemedicine process flow could lead to an improvement of the public health management, some limitations should be considered.

## Data Availability Statement

The original contributions presented in the study are included in the article/supplementary material, further inquiries can be directed to the corresponding author/s.

## Author Contributions

GS, MM-G, and SB contributed to the conceptualization of the manuscript and bibliographic review. MM-G, SB, and RD contributed to the writing. GR and OR contributed to the bibliographic review and writing of the manuscript. LG contributed to the bibliographic suggestions and the revision of the manuscript. CT, GS, and TP contributed to the supervision of the manuscript. All authors approved the final version of the manuscript for submission.

## Conflict of Interest

The authors declare that the research was conducted in the absence of any commercial or financial relationships that could be construed as a potential conflict of interest.
